# Properties of Protein Isolates from Marine Hydrobionts Obtained by Isoelectric Solubilisation/Precipitation: Influence of Temperature and Processing Time

**DOI:** 10.3390/ijms232214221

**Published:** 2022-11-17

**Authors:** Svetlana R. Derkach, Yuliya A. Kuchina, Daria S. Kolotova, Ludmila A. Petrova, Vasily I. Volchenko, Andrei Yu. Glukharev, Vladimir A. Grokhovsky

**Affiliations:** 1 Laboratory of Chemistry and Technology of Marine Bioresources, Murmansk State Technical University, 183010 Murmansk, Russia; 2 Department of Chemistry, Murmansk State Technical University, 183010 Murmansk, Russia; 3 Department of Food Production Technology, Murmansk State Technical University, 183010 Murmansk, Russia

**Keywords:** protein isolate, marine hydrobionts, alkaline solubilisation, isoelectric precipitation, functional properties

## Abstract

Protein isolates were obtained from marine hydrobionts by the method of isoelectric precipitation with a preliminary stage of protein alkaline solubilisation. Northern blue whiting was chosen as the raw material. Various technological modes of the solubilisation stage were used: the temperature of the reaction mixture was 4 or 20 °C, and the duration was 4 or 16 h. The yield of the product was 44–45% with a high content of the main component (protein) equal to about 95%. It has been shown that a decrease in the temperature and duration of the alkaline solubilisation stage provides the production of protein isolates with good technological properties, a low solubility, high swelling and high emulsifying ability, necessary for its use in the production of functional food products, including therapeutic and prophylactic effects. These technological properties are explained by a change in the composition and structure of the protein, the change being an increase in the content of essential amino acids and the proportion of α-helices in the polypeptide chain. The main patterns obtained will be used to obtain protein isolates from marine molluscs.

## 1. Introduction

Proteins are an important component of many foods that provide complete human nutrition. Currently, the consumption of modified protein products as ingredients of food systems is growing, which can not only increase their nutritional value, but also give them various functional properties, including a therapeutic and prophylactic effect in relation to many diseases [1,2]. At the same time, the use of modified protein products, among which hydrolysates, concentrates and protein isolates should be noted, contributes to the formation of the texture of the finished product due to the manifestation of such functional properties as solubility, gelation, emulsification, foaming, the ability to bind water and oil, etc.

Protein isolate is a highly concentrated protein fraction with a protein content of at least 90 wt.%, isolated from protein-containing raw materials of plant and animal origin. Protein isolates, as a rule, have indistinct organoleptic characteristics, lacking a pronounced colour, taste and smell. The protein in isolates is complete, that is, it contains all the essential amino acids, so protein isolates are used to create functional foods of an increased biological value [3]. To ensure all the necessary properties of the protein isolate, it is necessary to be exacting in the choice of raw materials for its production.

Currently, the industrial production of protein isolates is almost entirely based on the processing of protein-containing raw materials of plant origin [4]. However, plant proteins contain fewer essential amino acids and are less digestible than animal proteins.

Marine hydrobionts, such as molluscs and marine fish, are a source of food proteins, lipids and minerals. Sea fish is an excellent source of food proteins, lipids and minerals [5]. Increased demand has resulted in the depletion of traditionally exploited marine fish stocks, while low-value fish species are underutilised. This has led to an increase in the number of studies in the field of technologies for extracting fish proteins both from existing fishery objects and from new, previously unused raw material sources [6,7].

Blue whiting (*Micromesistius poutassou*) is a commercial fish of the cod family, ranking fifth in the world in terms of catch volumes (stocks are especially large in the Norwegian and Barents Seas); it is a low-fat fish with a high protein content (at least 16%). Blue whiting muscle tissue contains biologically complete proteins with an optimal amino acid composition. Therefore, blue whiting can be considered as a promising raw material source for the production of high-quality fish protein isolates (FPIs).

Various physical and chemical methods are widely used to obtain protein isolates, such as thermal extraction [8], sonication [9], homogenization [10], salting out [11], alkaline or acid solubilisation, isoelectric precipitation [12], enzymatic hydrolysis [13], etc.

Alkaline solubilisation followed by isoelectric precipitation (pH shift method) is the most common method due to its effectiveness. An alkaline (pH > 7) aqueous medium is used to extract proteins from the raw materials of plant and animal origin by dissolving them. At the same time, in an alkaline aqueous medium, the secondary structure of the protein changes, which increases its solubility [14]. Then, the pH of the aqueous medium is adjusted to the isoelectric point of the dissolved proteins; the total charge of macromolecules becomes equal to zero and the solubility drops sharply, which leads to the precipitation of the proteins. The protein isolates obtained in this way have all the functional properties necessary for the food industry [7].

In recent years, many works have been published on the use of the pH shift method to obtain FPIs from various fish species and their processing waste, such as Atlantic croaker [15], herring, cod and salmon [16], herring processing waste [17], rainbow trout [18], silver carp [19] and others. Works have been published in which the effect of process parameters on the yield and functional properties of FPIs has been studied. Thus, it was shown in [20,21,22] that protein isolates obtained upon solubilisation in an alkaline medium have better gel-forming properties compared to isolates obtained upon solubilisation in an acidic medium. An increase in the pH from 11.5 to 12.5 at the stage of alkaline solubilisation reduces the strength of the gel obtained from the FPI [23]. It has been shown [24] that the use of an ultrasound in combination with alkaline solubilisation makes it possible to increase the isolate yield and reduce the alkali consumption.

It should be noted that most studies in the literature are devoted to the study of the effect of alkaline dissolution pH on the functional properties of FPIs. At the same time, there are no studies on the effect of technological regimes of the alkaline solubilisation stage which significantly affect the yield and the properties of the FPIs. Therefore, the purpose of this work was to study the effect of the technological regime—the temperature and duration of the alkaline dissolution of the proteins—on the physicochemical (see Section 2.1) and functional (see Section 2.2) properties of the FPIs obtained from the muscle tissue of northern blue whiting. In our work, the task was to evaluate the effect of changing physicochemical properties, namely the amino acid composition (content of hydrophilic amino acids) and the secondary structure of the protein, on the functional properties of FPIs: the degree of swelling, solubility and emulsifying ability.

## 2. Results and Discussion

### 2.1. Physicochemical Properties of Protein Isolates

#### 2.1.1. Chemical Composition and Protein Yield

The FPIs obtained by the isoelectric solubilisation/precipitation method at different temperatures (t, °C) and duration (τ, h) of the alkaline solubilisation were a fine powder of a light beige colour, either completely odourless or with a slight fishy odour. Table 1 shows the chemical composition of FPIs and raw material, as well as the yield of protein obtained under different technological conditions.

The protein yield is an important technological process indicator. It can be seen that an increase in the duration of the alkaline solubilisation led to an increase in the protein yield to 44.5% for FPI 4-16 and 45.4% for FPI 20-16 compared to 44% for FPI 20-4; an increase in the temperature also led to an increase in the product yield from 44.5% for FPI 4-16 to 45.4% for FPI 20-16. The analysis of the data presented in Table 1 shows that the FPIs are characterised by a high protein content (more than 93 wt.%), a small amount of ash (no more than 1.2 wt.%) and no fat. An increase in the duration of the alkaline solubilisation stage led to an increase in the protein content of the isolate and a decrease in the moisture content. So, samples FPI 4-16 and FPI 20-16 contained 95.1% and 95.3% of protein, while sample FPI 20-4 contained only 93.8%. At the same time, the temperature changes did not have a noticeable effect on the chemical composition of the obtained isolates.

The absence of fat in all the FPI samples is due to its low content in raw materials, as well as the conditions for obtaining the isolates. In alkaline processing, soluble proteins are separated from lipids at high pH values and then removed during centrifugation [25]. A significant decrease (by 90%) in the amount of fat was noted during the alkaline treatment of pangasius fillets. Similar results were obtained during the extraction of proteins from substandard mackerel [26].

#### 2.1.2. Amino Acid Composition

The amino acid composition of raw material and FPI obtained under various conditions of alkaline solubilisation of fish proteins is presented in Table 2.

The experimental results showed that the technological conditions (temperature and time) of the alkaline solubilisation of the fish proteins affect the amino acid composition of the obtained FPIs. The main amino acids found in the FPI were glutamic acid, aspartic acid, leucine and lysine. The share of glutamic acid, aspartic acid and leucine increased compared to the raw material. However, the share of histidine and threonine decreased by two times, and that of glycine by three times, compared with the muscle tissue of northern blue whiting. Apparently, this is due to the fact that not all fish proteins rich in histidine, threonine and glycine are dissolved in an alkaline solution and then precipitated in the isoelectric precipitation stage. These data are consistent with the results published in [16,17], where the FPIs obtained from salmon, cod and herring fillet had a lower content of glycine compared to that in the original raw material. The authors suggested that one of the reasons is the loss of insoluble proteins rich in glycine.

The nutritional value of the protein is mainly determined by the content of essential amino acids. A high temperature and long duration of the alkaline solubilisation stage resulted in a low content of essential amino acids in the FPI. Thus, FPI 20-16, obtained by alkaline solubilisation at 20 °C for 16 h, contained 37.8 g/100 g of protein, which is significantly less than in the raw materials and FPI samples 4-16 and FPI 20-4, containing 39.6 and 39.3 g/100 g of protein of essential amino acids, respectively (Table 2). Apparently, a high temperature in combination with the duration of the solubilisation stage promotes, along with dissolution, the partial hydrolysis of the fish proteins to the polypeptide fragments. Polypeptide fragments containing essential amino acids are not precipitated in the isoelectric protein precipitation stage and are removed with the solution during the centrifugation.

#### 2.1.3. Secondary Structure of Proteins

The IR spectra of the proteins contained several main absorption bands: wide absorption bands at a frequency of 3400–3300 cm^−1^ (amide A) and 3000–2900 cm^−1^ (amide B), corresponding to the stretching vibrations of N–H bond atoms, and bands in the frequency range of 1700–1600 cm^−1^ (amide I, stretching vibrations of C=O and C–N bonds), 1575–1480 cm^−1^ (amide II, bending vibrations of N–H bonds and C–N stretching vibrations) and 1300–1230 cm^−1^ (amide III, stretching vibrations of the C–N bond) [16,28,29].

**Figure 1:**
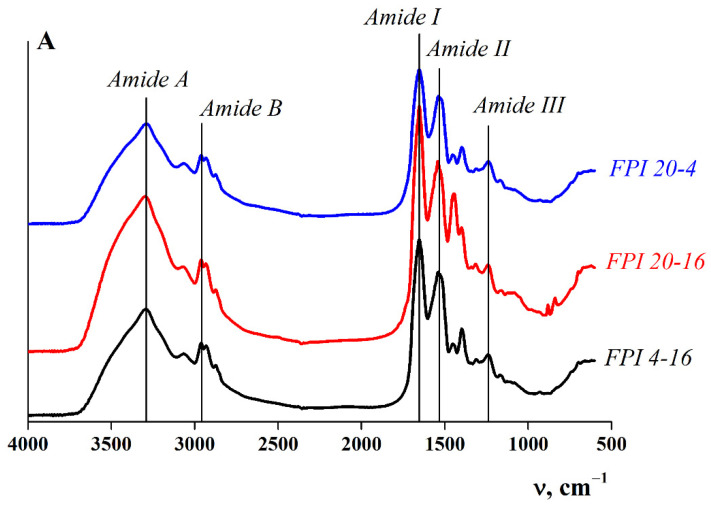
FT-IR spectra of fish protein isolates (FPIs) obtained under various conditions of alkaline solubilisation. FPI sample designations are given next to the spectra.

The IR spectra of the FPIs are typical for proteins and have absorption peaks at characteristic frequencies (Figure 1). The analysis of the IR spectra showed that changing the conditions (temperature and time) of the alkaline solubilisation stage during the preparation of the FPIs did not lead to a shift in the peaks.

An alkaline environment can cause changes in the secondary, tertiary and quaternary structure of proteins and their agglomerates [14]. At high pH values, protein macromolcules unfold due to an increase in the mutual repulsion forces in the polypeptide chains, so their hydrophobic and free sulphydryl (–SH) groups become available for an interaction. In addition, the reactivity of some amino acid residues, such as cysteine, increases in an alkaline environment, which leads to the involvement of cysteines in thiol–disulphide exchange reactions. Strong intermolecular interactions affect the secondary structure of the protein by changing the ratio between different conformational states of the protein molecule regions: β-turns, β-sheets, random coils and α-helices [30]. The amide I band is known to be the most sensitive to these changes.

**Figure 2:**
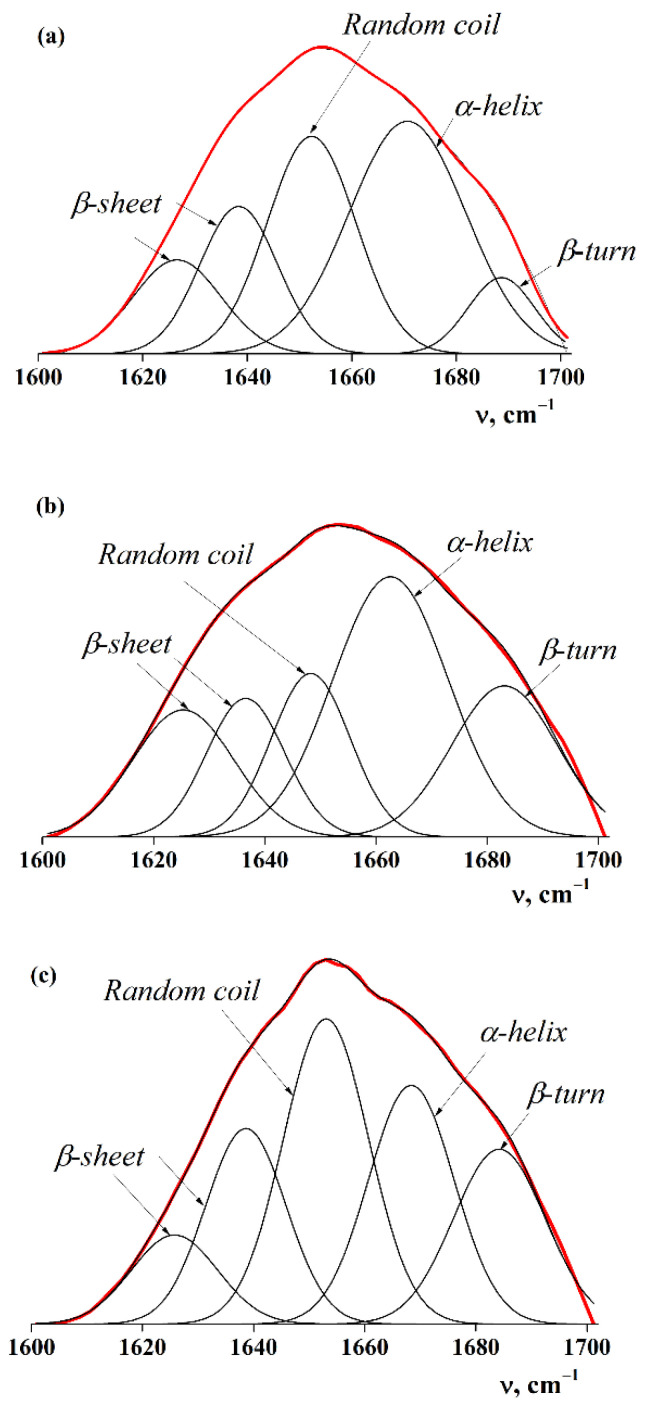
Decomposition of the amide I band (red line) into five spectral lines (black lines) corresponding to different conformations of protein molecules using a Gaussian distribution (R^2^ = 0.99) for fish protein hydrolysates: (**a**) FPI 4-16; (**b**) FPI 20-16; and (**c**) FPI 20-4.

To obtain the quantitative information, we decomposed the amide I band into several components using a Gaussian distribution using the OriginPro 9.0 graphics program (Figure 2). The quantitative contribution of the macromolecular chains in different conformations was determined as the ratio of the integrated intensity of the corresponding band to the integrated intensity of the amide I band before the decomposition (Table 3).

The data in Table 3 show that changing the conditions of the alkaline solubilisation step in obtaining the FPIs causes changes in the secondary structure of the proteins. An increase in time led to a 1.5-fold decrease in the number of α-helices, from 36.5% (for FPI 20-4) to 23.8% (for FPI 20-16). An increase in temperature also led to a 1.6-fold decrease in the number of α-helices, from 38.1% (for FPI 4-16) to 23.8% (for FPI 20-16). The lowest helix content was demonstrated by FPI 20-16 obtained at 20 °C for 16 h.

It is known that α-helices are responsible for the native structure of a protein and the manifestation of its functional properties, for example, the ability to gel. We used the ratio of the helix content to the coil content (Table 3) for a quantitative assessment of the components; it is equal to the ratio of the integrated intensities (A) of the peaks at 1668 and 1650 cm^−1^, respectively (A_1668_/A_1650_) [32]. FPI 20-4 showed the highest A_1668_/A_1650_ value, equal to 2.3. Perhaps, at temperatures not exceeding 20 °C, the processing time has a stronger effect on the change in the native structure of the protein rather than the temperature.

The next section will show how the physicochemical properties of the fish protein isolate, such as the content of essential amino acids and the secondary structure that characterise the composition and structure of the protein, affect its functional properties: the ability to swell, the solubility and the stabilisation of the emulsion systems.

### 2.2. Functional Properties of Protein Isolates

#### 2.2.1. Degree of Swelling and Solubility

Solubility is an important characteristic of FPIs that determines their further use. The solubility of proteins in water is due to the presence of hydrophilic groups (hydroxyl, sulphydryl and amide) on the surface of the protein molecule, belonging to polar amino acids and capable of binding water [33]. The solubility of FPIs in water depends on their physicochemical properties, for example, the structure of macromolecules, and external factors such as the pH value, the presence of electrolytes, temperature, etc. [7,16].

The initial stage of dissolution consists of the diffusion of water molecules into the volume of the biopolymer and an increase in its volume and mass. The quantitative characteristic of this process, the degree of swelling (S_W_, g/g), shows how many times the mass of the biopolymer increases upon contact with the solvent.

**Figure 3:**
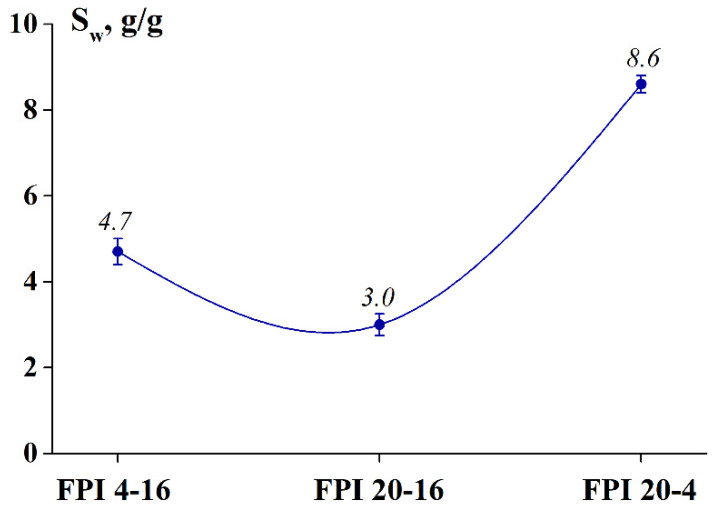
Degree of swelling of fish protein isolates (FPIs) obtained from northern blue whiting under various conditions of alkaline solubilisation.

The lowest degree of swelling, equal to 3.0 g/g (Figure 3), was found for FPI 20-16 obtained by alkaline solubilisation at a high temperature (20 °C) and a long treatment time (16 h). Reducing the temperature to 4 °C and treatment time to 4 h led to an increase in the degree of swelling by 1.5 times for FPI 4-16 and 2.8 times for FPI 20-4.

**Figure 4:**
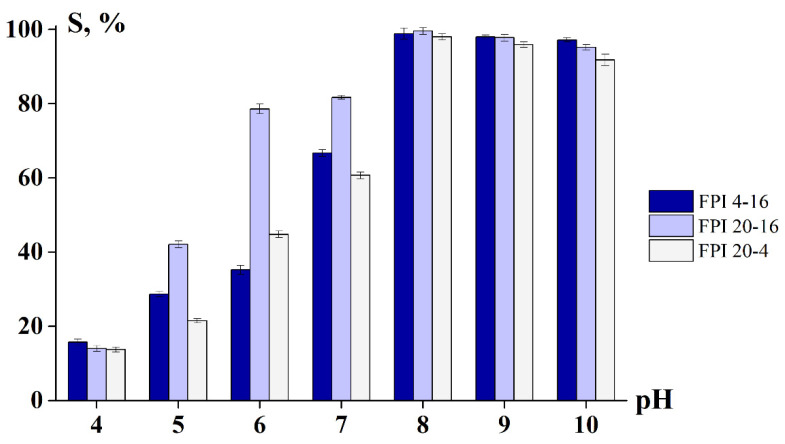
Influence of pH on the solubility of fish protein isolate (FPI). Sample designations are shown on the graph.

The low degree of swelling of FPI 20-16 is most likely due to its higher solubility (Figure 4), which in turn is due to the low content of α-helices (see Table 3), which are responsible for the native properties of the protein. It is possible that not only the swelling of the FPI 20-16 sample occurred during the experiment, but also its dissolution. Accordingly, the high degree of swelling of FPI 20-4 and FPI 4-16 is determined by the high content of α-helices (see Table 3) and polar (hydrophilic) amino acids (see Table 2). The higher the degree of swelling of FPI 20-4 correlates with a higher content of hydrophilic amino acids (52.1 g/100 g) compared to FPI 4-16. During the swelling, water molecules penetrate the protein and interact with its polar groups. The dense packing of the polypeptide chains is loosened, facilitating a further dissolution.

Solubility is an important property of FPIs, as it affects other functional properties, such as the emulsifying ability, foaming and thermal properties [34]. Figure 4 shows the solubility of the obtained FPIs as a function of the pH value. The highest solubility (up to 96–99%) is observed in the pH range around 8 to 9, and the lowest solubility (14–40%) is observed in the pH range of the protein isoelectric point, around 4 to 5.

The data obtained correlate with the general patterns of protein solubility [35,36,37]. At the isoelectric point (pI), proteins reach the zwitterion structure and macromolecules are characterised by a zero charge, which leads to the formation of protein aggregates and limits the protein solubility to minimum values [36,37]. It should be noted that FPI 20-16, obtained at a high temperature (20 °C) and long duration (16 h) of alkaline solubilisation, is characterised by a high solubility, significantly exceeding the solubility of other isolate samples at pH 5 to 7. A high solubility correlates with a low content of macromolecule sites in the conformation of α-helices (see Table 3).

#### 2.2.2. Emulsifying Ability and Dispersion of Concentrated Emulsions

**Figure 5:**
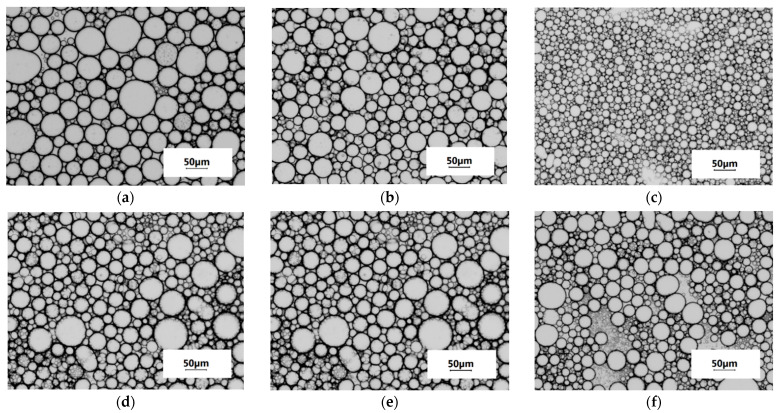
Microphotographs of concentrated emulsions stabilized with FPI 4-16 (**a**–**c**) and FPI 20-4 (**d**–**f**). C(FPI), % (*w*/*v*): 1.0 (**a**,**d**); 2.0 (**b**,**e**); and 5.0 (**c**,**f**). Magnification 10×.

The emulsifying ability (X) determines the possibilities of using FPI in the creation of emulsion foods. Table 4 shows the characteristics of oil/water emulsions stabilised with FPI 4-16 and FPI 20-4. With an increase in the FPI concentration from 1.0% to 5.0%, the initial sedimentation rate (υ_0_) of the emulsion (φ_0_ = 50%) decreased sharply, which indicates an increase in the sedimentation stability. With an increase in the stabiliser concentration, the droplet size of the concentrated emulsions (φ = 64–71%) obtained by the sedimentation of the original emulsions decreased from 41 to 18 μm in the case of FPI 4-16, and from 31 to 25 µm in the case of FPI 20-4 (Table 4). Figure 5 illustrates the micrographs of the emulsions.

**Figure 6:**
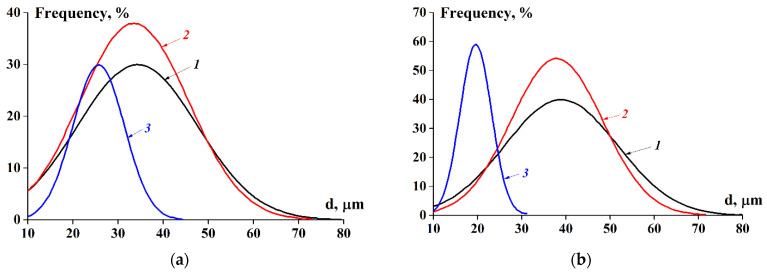
Droplet size distribution for concentrated emulsions stabilised with FPI 20-4 (**a**) and FPI 4-16 (**b**). C(FPI), % (*w*/*v*): 1—1.0; 2—2.0; and 3—5.0.

Figure 6 shows the typical droplet size distribution curves for the concentrated emulsions stabilised with the FPIs. The emulsions with stabiliser concentrations of 1.0% and 2.0% (*w*/*v*) show a broad monomodal distribution peak in the range from 10 to 80 µm, with a maximum in the region of 30–40 µm. The emulsions with a stabiliser concentration up to 5% (*w*/*v*) are characterised by the presence of one narrow distribution peak in the range from 10 to 30–40 µm, with a maximum in the region of 18–25 µm. The average diameter of the droplets is given in Table 4.

With an increase in the concentration of the stabiliser FPI 4-16 or FPI 20-4, an increase in the emulsifying ability was observed, which correlates with an increase in the dispersion of the emulsions (see Table 4), confirming the well-known pattern [38]. FPI 4-16 and FPI 20-4 demonstrated very similar values of their emulsifying ability at the same concentration, which is probably due to the same solubility [39] of these protein isolates (see Figure 4).

Thus, the analysis of the obtained results shows that a low solubility, a high swelling and an emulsifying ability, which are so in demand in food technologies, are observed in the FPIs with a high content of essential amino acids and a protein secondary structure with a predominance of α-helices.

## 3. Materials and Methods

### 3.1. Raw Materials

The FPI was obtained from the muscle tissue of northern blue whiting (*Micromesistius poutassou*). The fish were caught in the Central–Eastern region of the Atlantic, frozen and delivered to the port of Murmansk, where they were stored for 1 month before research began at a temperature no higher than −18 °C.

All the chemical reagents used in the work were of an analytical grade (*Pro Analysi*).

### 3.2. Preparation of Fish Protein Isolates

**Figure 7:**
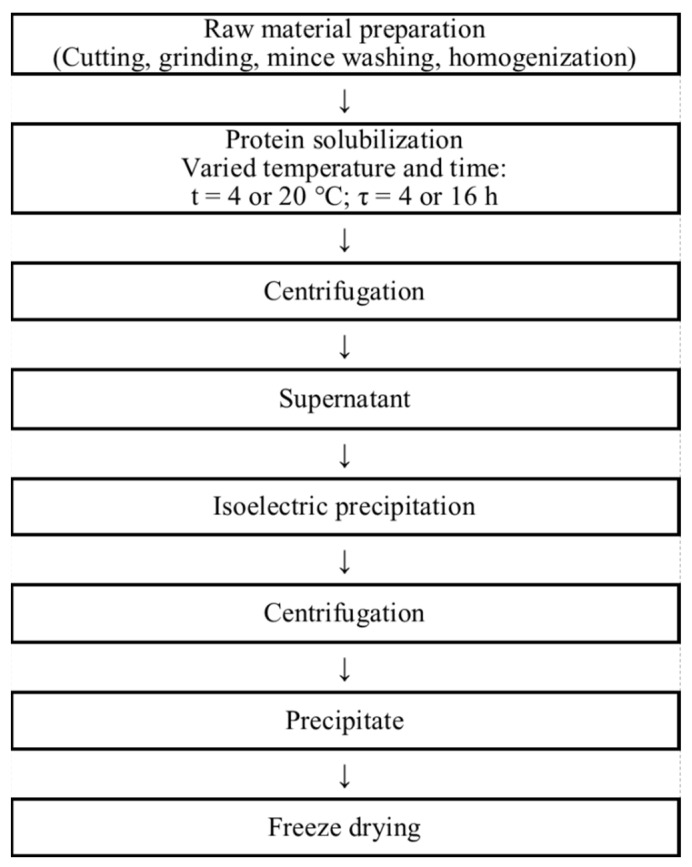
Technological scheme for obtaining protein isolate.

The FPIs were recovered from blue whiting muscle tissue by the isoelectric solubilisation/precipitation method according to [7], with minor changes. Figure 7 illustrates the FPI processing stages. The fish were defrosted and cut into skinned fillets. The fish fillets were ground to mince (particle size 3 mm × 3 mm), then washed with water twice as follows. The minced fish were mixed with distilled water in a mass ratio of 1:2, stirred at room temperature for 5 min and filtered through four layers of muslin cloth. The fish mince was washed in order to remove water-soluble substances that form an unpleasant fishy odour. These substances include free amino acids, peptides and volatile bases (ammonia, di- and trimethylamines).

After washing, the minced fish was homogenized with a 2 M NaOH solution at a 1:4 mass ratio using an Ace AM-1 laboratory homogenizer (Nihonseiki Kaisha Ltd., Akita City, Japan) at 5000 rpm and at 4–5 °C for 2 min, then the solubilisation reaction was allowed take place with stirring under various conditions: (1) at 4 °C for 16 h (sample FPI 4-16); (2) at 20 °C for 16 h (sample FPI 20-16); and (3) at 20 °C for 4 h (sample FPI 20-4). The pH value of the reaction medium was 9.5 ± 0.5.

The solubilisation reaction was followed by centrifugation at 3500 rpm and at 4 °C for 10 min using a refrigerated centrifuge (RS-6MTs equipped with a RK4x750M rotor, Russia). The centrifugation resulted in two layers: a fish protein solution and insoluble fractions (insoluble proteins, cell membranes, lipids, etc.).

Then, the pH of the fish protein solution (supernatant) was adjusted to 5.5 ± 0.1 with a 2 M HCl solution for the isoelectric precipitation of the fish proteins. It is known that the precipitation of fish proteins is ensured by adjusting the pH to the isoelectric point of the myofibrillar proteins, around 5.2 to 5.5 [40]. The precipitated proteins (FPI) were dehydrated by centrifugation at 3500× *g* rpm and 20 °C for 5 min.

The recovered FPIs were dried in a FreeZone freeze dryer (Labconco, Kansas City, MO, USA) at −50 °C and a residual pressure of 2.8–4.5 Pa. Freeze drying makes it possible to form a porous sample structure without shrinkage, while maintaining the original shape and size of the protein molecules; it provides a fast and almost complete rehydration of the samples. The obtained FPIs were stored at 5 ± 1 °C until further use.

The protein yield (B, %) was calculated using the formula [41]:

(1) $$B=\frac{P_{\mathrm{FPI}\;}\cdot m_{\mathrm{FPI}}}{P_{F}\;\cdot {\;m}_{F}}\;\cdot \;100,$$

where $m_{F}$ is the mass of the wet raw materials (skinned fillets), kg; $m_{\mathrm{FPI}}$ is the mass of dried FPI, kg; P_F_ is the mass fraction of the protein in the wet raw materials, %; and P_FPI_ is the mass fraction of the protein in the dried FPI, %.

### 3.3. Chemical Composition of Raw Materials and Fish Protein Isolates

The moisture, fat, total nitrogen and mineral content of the raw materials and FPI was determined according to the standard methods [42]. The moisture content in the samples was determined after drying them to a constant weight at t = 105 ± 5 °C. The amount of fat was determined by the Soxhlet method (solvent extraction), the total nitrogen by the Kjeldahl method and the mineral substances by burning samples in a muffle furnace at t = 550 ± 10 °C.

### 3.4. Amino Acid Composition

The amino acid composition of the FPI was determined by high-performance liquid chromatography (HPLC). The method is based on the chromatographic separation of amino acids modified with o-phthalaldehyde and β-mercaptoethanol, followed by a registration with a spectrofluorimetric detector [43,44]. A liquid chromatograph (model LC-10A, Shimadzu, Kyoto, Japan) and a column (model SUPELCOSIL LC-18, 4.0 mm × 30 cm, 5 µm) were used. The gradient separation was carried out with a binary eluent (acetonitrile/0.05 M sodium acetate solution), an eluent flow rate of 1.5 mL/min and a column temperature of 35 °C. The peaks were recorded using a spectrofluorimetric detector (model RF-10 AXL) with an excitation wavelength of 350 nm and an emission wavelength of 450 nm. The column was calibrated using standard amino acid samples from Sigma-Aldrich, St. Louis, MO, USA.

### 3.5. Secondary Protein Structure

Fourier-transform infrared (FT-IR) spectroscopy was used to evaluate the secondary structure of the fish protein. The absorption spectra were recorded on an FT-IR spectrophotometer (model IRTracer-100, Shimadzu, Kyoto, Japan) in the frequency range from 4000 to 800 cm^−1^, at a resolution of 4 cm^−1^; the number of scans was 250. The sample for the measurements was a mixture of FPI and KBr in a mass ratio of 1:60. The mixture was dissolved in distilled water, then dried in a freeze dryer at −53 °C and a residual pressure of 2.4–2.6 Pa for 8–10 h [45]. The dried mixture was additionally kept at a temperature of 65 ± 5 °C for 12 h. Then, the mixture (m = 150 mg) was compressed under a pressure of 650 kgf/cm^2^ into a tablet. The IR spectra were taken immediately after the pressing. In order to minimise the effect of the traces of moisture (water vapor), the spectrum of water vapor was subtracted from the obtained spectra of the studied samples.

The IR spectrum in the absorption region of the amide I band (1600–1700 cm^−1^) was processed using the OriginPro 9.0 graphics program (OriginLab Corporation, Northampton, MA, USA). The secondary structure of the protein was analysed by the second derivative method. To obtain the quantitative information, the amide I band was decomposed into several components using a Gaussian distribution [31,34]. The quantitative contribution of each conformation (component) [46] of the secondary structure was determined as the ratio of the integrated intensity of the corresponding band to the total integrated intensity of the amide I band before the decomposition.

### 3.6. Swelling Ability

The swelling ability of the FPI was determined by the gravimetric method. The method is based on measuring the amount of water absorbed by the sample during flooding. We used the technique proposed in [47] with minor modifications. The FPI and distilled water in a mass ratio of 1:20 were placed in centrifuge tubes; the contents of the tubes were thoroughly mixed on a shaker (model LAB PU-02, LOiP, St. Petersburg, Russia) for 2 h at a speed of 300 rpm. Then, the test tubes with the FPI were left for 6 h at room temperature (20–25 °C) for swelling. Any excess water was removed using a laboratory centrifuge (model OPn-8) at 8000× *g* rpm for 30 min. Then, the contents of the test tubes were weighed.

The degree of swelling (S_w_, g/g) was calculated by the formula:

(2) $$S_{w}=\frac{m_{1}}{m_{2}},$$

where m_1_ is the mass of the sample after swelling and centrifugation (g) and m_2_ is the mass of the initial dry sample of the FPI taken for analysis (g).

### 3.7. Solubility

The solubility of the FPI was determined by the procedure described in [48]. A series of mixtures of FPI with water (5 wt.%) at different pH values from 3.0 to 10.0 was prepared, and solutions of HCl (2 M and 0.1 M) and NaOH (2 M and 0.1 M) were used to adjust the pH. The mixtures were thoroughly mixed at room temperature for 30 min and then kept at 5 ± 1 °C for 12 h. The undissolved fractions were removed using a laboratory centrifuge (model OPn-8) at a rotor speed of 8000× *g* rpm for 5 min. In the dried supernatant, the total nitrogen was determined by the Kjeldahl method.

The solubility (S, %) of FPIs was determined by the formula:

(3) $$S=\frac{N_{T}}{N_{T_{\mathrm{FPI}}\;}}\;\cdot \;100,$$

where $N_{T}$ is the amount of total nitrogen in the dried supernatant (wt.%) and $N_{T_{\mathrm{FPI}}}$ is the amount of total nitrogen in the FPI (wt.%).

### 3.8. Emulsifying Ability

Original oil/water emulsions were obtained by dispersing the oil and water phases in a volume ratio of 50:50 (emulsion concentration φ_0_ = 50%) at room temperature using a disperser (model T25 Digital Ultra-Turrax, IKA, Staufen, Germany) at 8000 rpm for 10 min. As a dispersion medium, aqueous solutions of FPI (FPI 4-16 and FPI 20-4) with concentrations of 1.0%, 2.0% and 5.0% (*w*/*v*) and a pH value = 8 were used, and refined deodorised sunflower oil was used as the oil phase. Then, the concentrated oil/water emulsions stabilised with the FPI were obtained by reverse sedimentation [49]. To study the sedimentation stability, the original emulsions (φ_0_ = 50%) were placed in measuring cylinders and the volume (V_water,τ_, cm^3^) of the aqueous phase separated during the reverse sedimentation was measured for 72 h. The initial sedimentation rate (υ_0_, cm^3^/h) was determined from the sedimentation kinetic curves.

The emulsion concentration (φ, %) at time τ (h) was determined by the formula:

(4) $$\varphi_{\tau }=\frac{V_{\mathrm{oil},0}}{V_{\mathrm{emulsion}}}\cdot 100\;\%=\frac{V_{\mathrm{oil},0}}{V_{\mathrm{oil},0}{+V}_{\mathrm{water},0}-{\;V}_{\mathrm{water},\tau }}\cdot 100,$$

where V_emulsion_ is volume of the emulsion, cm^3^; V_oil,0_ is the initial volume of the oil (dispersed) phase in the emulsion, cm^3^; V_water,0_ is the initial volume of the aqueous phase in the emulsion, cm^3^; and V_water,τ_ is the volume of the aqueous phase separated at time τ (h) during the reverse sedimentation, cm^3^.

The emulsifying ability (X, %) of the FPI was determined 72 h after the preparation of the original emulsion according to the formula:

(5) $$\;X=\frac{V_{\mathrm{emulsion}}}{V_{\mathrm{oil},0}{+V}_{\mathrm{water},0}}=\frac{V_{\mathrm{oil},0}{+V}_{\mathrm{water},0}-{\;V}_{\mathrm{water},72h}}{V_{\mathrm{oil},0\;}{+V}_{\mathrm{water},0}}\;\cdot \;100,$$

where V_water,72h_ is the volume of the aqueous phase separated 72 h after the preparation of the original emulsion, cm^3^.

The droplet size of the concentrated emulsions obtained by the sedimentation of the original emulsions was determined by optical microscopy at a 10× magnification. A light microscope (model CX 43, Olympus, Tokyo, Japan) equipped with a digital camera and ToupView software (ToupTek Photonic, Hangzhou, China) were used. Droplet size distribution diagrams were built using Origin Pro 9.0. The average diameter (d, μm) of the droplets was calculated by the formula:

(6) $$d=\frac{\sum d_{i}}{n},$$

where d_i_ is the droplet diameter, µm and n is the number of droplets (~100 pieces).

### 3.9. Statistical Analysis

The measurements were carried out at least three times; the obtained data were subjected to a one-way analysis of variance using Origin Pro 9.0. Differences between the mean values were considered significant at *p* < 0.05.

## 4. Conclusions

The general patterns of obtaining protein hydrolysates from marine hydrobionts were considered. At present, the muscle tissue of molluscs, underused marine fish and waste from their processing are used as raw materials. The FPIs were obtained from the muscle tissue of northern blue whiting by the isoelectric precipitation method with a preliminary stage of alkaline solubilisation. The effect of the temperature and duration of the alkaline solubilisation of the fish proteins on the physicochemical and functional properties of the FPI was determined. All the FPIs obtained at temperatures of 4 and 20 °C and with processing times of 4 and 16 h showed a high protein content of up to 95% and no fat. The protein yield is 44.0–45.4% of crushed wet raw materials.

The experimental data show that the alkaline solubilisation conditions affect the amino acid composition, the secondary structure of the proteins and the solubility of the FPI. An increase in time from 4 to 16 h (at 20 °C) leads to a partial hydrolysis of the protein molecule and a change in the secondary structure of the protein, this being a decrease in the number of α-helices. At the same time, the yield of essential amino acids decreases, and the solubility at neutral pH values increases. A decrease in the temperature from 20 to 4 °C, on the contrary, leads to an increase in the content of essential amino acids in the protein, an increase in the number of α-helices and a decrease in the solubility at neutral pH values.

The solubility of the FPIs at a pH value above 8 is approximately the same and is in the range of 95–100%. The FPIs have a high emulsifying ability (65–75%), which increases as the concentration of the fish isolate in the emulsion increases. The emulsifying ability does not depend on the conditions for obtaining the isolates.

The analysis of the physicochemical and functional properties of the FPIs indicates that FPI 4-16, obtained by alkaline solubilisation at 4 °C for 16 h, has the best properties. It should be noted that FPI 20-4, obtained by alkaline solubilisation at 20 °C for 4 h, is practically not inferior to it in terms of the basic properties. In the case of planning the industrial production of the FPI from the muscle tissue of northern blue whiting, FPI 20-4 is certainly a more promising object, since it can be obtained at room temperature without an energy-consuming cooling of the system.

Further research will be aimed at expanding the raw material base for the production of protein isolates from marine hydrobionts. The regularities obtained in this work will be used to develop technological regimes for obtaining protein isolates from the soft tissues of marine molluscs (mussels), seen in aquaculture products that are actively developing at the present time.

## Figures and Tables

**Table 1:** Chemical composition of raw material and FPIs obtained under different temperatures and duration of alkaline solubilisation.

Samples	Solubilisation Conditions		Moisture Content ω_M_, %	Total Nitrogen N_T_, %	Protein * P, %	Fat, ω_F_, %	Ash, ω_A_, %	Protein Yield, B, %, *w*/*w*
	t, °C	τ, h						
Muscle tissue of northern blue whiting	−	−	81.1 ± 2.4	2.6 ± 0.1	16.3 ± 0.6	0.3 ± 0.2	1.4 ± 0.2	−
FPI 4-16	4 ± 1	16	3.9 ± 0.1	15.2 ± 0.1	95.1 ± 0.2	−	1.0 ± 0.1	44.5 ± 1.0
FPI 20-16	20 ± 1	16	3.8 ± 0.1	15.3 ± 0.1	95.3 ± 0.2	−	0.9 ± 0.1	45.4 ± 1.0
FPI 20-4	20 ± 1	4	4.9 ± 0.2	15.0 ± 0.1	93.8 ± 0.3	−	1.2 ± 0.1	44.0 ± 1.0

* The mass fraction of protein was calculated as P = N_T_ × 6.25 (6.25 is the conversion factor for the amount of nitrogen per protein). The nitrogen to protein conversion factor was selected in accordance with the EU Council Directive on Nutrition Labelling of Foods (90/496/EEC).

**Table 2:** Amino acid composition (amino acid content, g/100 g of protein) of raw material and FPI obtained under various conditions (temperature and duration) of alkaline solubilisation.

Amino Acids	Muscle Tissue of Northern Blue Whiting [27]	FPI 4-16	FPI 20-16	FPI 20-4
Glutamic acid (HAA)	13.1	16.3	16.2	16.6
Aspartic acid (HAA)	9.0	9.2	9.3	9.6
Leucine (EAA)	8.0	9.0	8.9	8.9
Lysine (EAA, HAA)	9.0	8.6	8.1	8.7
Arginine (HAA)	5.8	5.4	5.4	5.1
Alanine	6.5	5.4	5.3	5.3
Isoleucine (EAA)	5.2	5.1	5.0	5.0
Phenylalanine (EAA)	4.1	4.8	4.6	4.7
Valine (EAA)	5.5	4.7	4.7	4.7
Methionine (EAA)	3.1	4.6	3.8	4.4
Tyrosine	2.4	4.3	4.1	4.2
Serine (HAA)	4.1	4.1	4.0	4.2
Glycine (HAA)	9.6	3.1	2.8	3.1
Threonine (EAA, HAA)	5.2	2.8	2.7	2.9
Histidine (HAA)	3.9	2.0	1.8	1.9
Proline	2.5	2.4	2.2	2.5
EAA, total	40.1	39.6	37.8	39.3
HAA, total	59.7	51.5	50.3	52.1

EAA: essential amino acids; HAA: hydrophilic amino acids (hydrophilic/polar amino acids).

**Table 3:** Content (%) of the amide I components for fish protein isolates (FPIs) obtained under various conditions of alkaline solubilisation.

Conformational State	Wave Number ν, cm^−1^ [31]	FPI 4-16	FPI 20-16	FPI 20-4
β-Turn/β-sheet	1625–1626	11.7	9.0	15.7
	1636–1638	16.1	18.3	13.2
Random coil	1649–1652	27.1	30.6	15.7
α-Helix	1664–1668	38.1	23.8	36.5
β-Turn/β-sheet	1684–1688	7.0	18.3	18.9

**Table 4:** Characteristics of oil/water emulsions stabilised with different concentrations of FPIs.

Characteristic	C(FPI 4-16), % (*w*/*v*)			C(FPI 20-4), % (*w*/*v*)		
	1.0	2.0	5.0	1.0	2.0	5.0
υ_0_, cm^3^/h	1.63 ± 0.09	0.86 ± 0.05	0.03 ± 0.02	1.55 ± 0.09	0.73 ± 0.05	0.21 ± 0.04
X, %	67.0 ± 0.2	68.5 ± 0.1	85.1 ± 0.5	68.7 ± 0.2	73.8 ± 0.6	75.5 ± 0.5
φ (concentrated emulsion), %	0.74 ± 0.02	0.71 ± 0.04	0.64 ± 0.04	0.71 ± 0.02	0.68 ± 0.01	0.68 ± 0.02
d (concentrated emulsion), μm	41 ± 3	35 ± 2	18 ± 2	31 ± 2	30 ± 2	25 ± 5

## Data Availability

Not applicable.
